# The advantage of parallel selection of domestication genes to accelerate crop improvement

**DOI:** 10.1186/s13059-018-1537-7

**Published:** 2018-09-28

**Authors:** Martha Rendón-Anaya, Alfredo Herrera-Estrella

**Affiliations:** 1 Department of Plant Biology, Uppsala BioCenter, Swedish University of Agricultural Sciences, PO Box 7080, SE-750 07 Uppsala, Sweden; 2 Laboratorio Nacional de Genómica para la Biodiversidad, Centro de Investigación y de Estudios Avanzados del IPN (Cinvestav), 36821 Irapuato, Guanajuato, Mexico

## Abstract

A recent study identifies a locus controlling seed dormancy – a key trait of the ‘domestication syndrome’ – that has been selected for in parallel across multiple crop families.

## Introduction

**Fig. 1:**
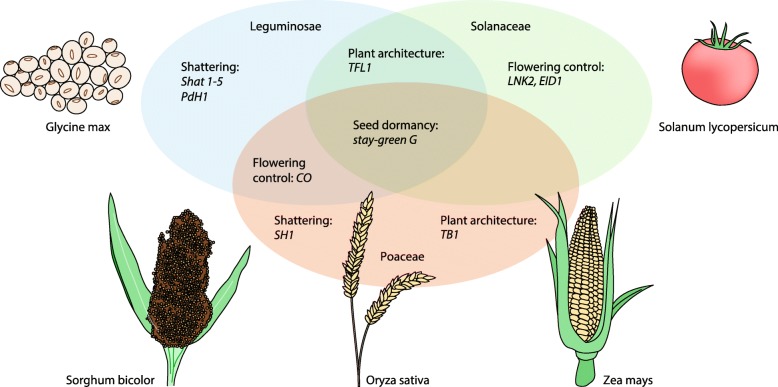
Examples of parallel selection of protein-coding genes across plant families. Depicted are: maize, *Z. mays*; rice, *O. sativa*; sorghum, *S. bicolor*; soybean, *G. max*; and tomato, *S. lycopersicum*

Ten to twelve thousand years ago, in different regions around the world, humans launched an intriguing ‘co-evolutionary experiment’ – the domestication of crop plants and farm animals. In the case of crops, domestication was expected to generate more-productive plants, better adapted to different agro-ecosystems and easier to harvest and/or producing edible products that were safer to consume. Although the species and locations vary, domestication events are typically associated with the series of morphological changes that collectively are known as the ‘domestication syndrome’. This encompasses cultivars with large non-dispersing seeds, uniform germination, photoperiod insensitivity, reduced branching and several nutrition-related phenotypes. The emergence of such phenotypic traits of morpho-agronomic relevance shared by different crops prompts a question regarding whether similarities in human demands regarding cultivation, harvest and consumption led to widespread convergent artificial selection of genes (Fig. 1). A recent study by Wang and colleagues [1] shed lights on this interesting issue.

Traditional genetic screenings and large-scale genomic studies have allowed the identification of a number of domestication-related genes that, in many cases, are particular to each domestication history, even within the same plant genus [2–4]. More recently, the combination of expression quantitative trait loci (eQTLs) and metabolome-based genome-wide association studies (mGWAS) has shown that domestication has rewired several metabolic and transcriptional networks [5]. In addition, the fact that genetic backgrounds can have pervasive effects on gene interactions, such as dominance, pleiotropy and epistasis [2], and considering the dynamism of genomic introgression between cultivars and their wild relatives, it becomes challenging to conclude with any confidence how many paths are necessary to achieve similar domestication phenotypes.

## Convergence of domestication traits and their genetic background

Domestication has occurred across varied cultures and geography. Even though there has been a strong convergence in terms of the evolution of domestication-related traits in different crop families, there are few examples where one single gene has been equally affected by artificial selection in distantly related species (*Shattering1* in cereals [6]). More often, parallel selection has occurred within a single plant species or genus. Broadly, domestication could be considered as an experiment replicated multiple times, given that domesticated crops tend to experience selective pressures that drive them towards shared phenotypic shifts [7]. This scenario implies that, if genes were selected in parallel, the number of genetic solutions to the challenge of domestication would be under constraint. However, a recent analysis of domestication – considering it an experimental evolution assay – [3] found no evidence for parallel selection events either between species (maize vs rice) or within species (two domestication events within beans).

## From seed coat pigmentation to dormancy – Two traits, one gene in common

Seed dormancy delays and desynchronizes germination under unsuitable environmental conditions for plant growth. In the wild, this is a useful strategy that promotes seed dispersal and warrants that at least some seeds will germinate and survive when conditions are favourable. In domesticated cultivars, however, it is desirable to have uniform germination to ensure maturity and crop management by farmers. Thus, the loss of seed dormancy has been a crucial trait selected for during crop domestication. Different studies have associated dozens of QTLs to seed dormancy in cereals and legumes, which explain a rather discrete amount of phenotypic variation.

In a recent report by Wang and collaborators [1], a stay-green locus, the *G* gene (*GmG*) encoding a CAAX amino-terminal protease and controlling the pigmentation of the seed coat, was found to be strongly associated with the loss of seed dormancy in soybean. Not only does this gene display signatures of strong selection in soybean, but its orthologs in rice (*OsG*) and tomato (*SolyG*) are also encoded within genomic regions with signatures of selective sweeps, as seen by tests of *F*_ST_, cross-population composite likelihood ratio (XP-CLR) and extended haplotype homozygosity (EHH). Based on genome resequencing of 176 rice accessions and a large available collection of wild and domesticated cultivars, it was observed that two groups of haplotypes in the population differentiate wild (group I) from domesticated *Indica* and *Japonica* accessions (group II). Similarly, in a large collection of 360 resequenced accessions, it was demonstrated that cherry (CER) and big-fruited (BIG) tomato cultivars displayed a non-synonymous single-nucleotide polymorphism (SNP; *C* allele) that differentiated them from the *G* allele present in the wild *Solanum pimpinellifolium*.

Experimental validation of the role of the *G* locus in seed dormancy was achieved using transgenic soybean and rice lines – that is, *GmG* introduced into a *g* allele soybean cultivar (DN50), *OsG* introduced into a *g* allele cultivated rice line (ZH11), a CRISPR knockout of *Osg* in ZH11, overexpression of two *OsG* alleles from *Oryza rufipogon* and *Osg* in ZH11. In all these combinations, the *G* allele was confirmed to cause a strong dormancy phenotype compared with the *g* form of the locus. Furthermore, *GmG* introduced in *Arabidopsis atg* mutants complemented the fast-germinating phenotype, showing that this gene is also involved in seed dormancy in *Arabidopsis*.

The function and possible interactors of the G protein were evaluated by means of yeast two-hybrid assays. *Arabidopsis AtG* was found to interact with two key enzymes of the abscisic acid (ABA) biosynthetic pathway – AtNCED3 and AtPSY. Both ABA and several carotenoids were observed to be more abundant in AtG than atg lines, as well as in transgenic soybean lines overexpressing *GmG* compared with *Gmg* cultivars.

The results of this study confirm the role of ABA in seed dormancy as previously reported, but, more importantly, they show for the first time that the same protein-coding gene has been targeted by artificial selection in three distant crop families, Leguminosae, Poaceae and Solanaceae (Fig. 1), and possibly even in Brassicaceae, given its contribution to seed dormancy in *Arabidopsis*.

## Future directions

As plant breeding has traditionally been based on phenotype-targeted selection, it becomes crucial to dissect the genetic elements behind relevant morpho-agronomic traits to improve crops with great precision and efficiency. Furthermore, crop breeding does not rely uniquely on direct selection, but also on the effects of linked loci that hitchhike during introgression events, such as metabolic genes hitchhiking with fruit weight genes as large tomato fruits were selected, or metabolic genes from inedible green-fruited species dragged by resistance genes from wild relatives [5].

In the past few years, our understanding of crop domestication has evolved from the view of a few large-effect loci to polygenic interactions that alter complex networks. In this context, the results reported by Wang and colleagues provide an excellent candidate for targeted breeding, as the introduction of a limited number of *G*/*g* alleles identified in distant plant families could potentially decrease the seed dormancy phenotype in other crops. Thus, high-throughput molecular breeding combined with precise genome editing should accelerate crop improvement and even, perhaps, domestication of plant species capable of growing under unforeseen environmental conditions, which could be crucial in the near future when facing changeable climatic conditions.

## References

[1] Wang M, Li W, Fang C, Xu F, Liu Y, Wang Z, et al. Parallel domestication of a dormancy gene in crops from multiple families. Nat Genet. 10.1038/s41588-018-0229-2. 10.1038/s41588-018-0229-2 30250128

[2] Stitzer Michelle C., Ross-Ibarra Jeffrey (2018). Maize domestication and gene interaction. New Phytologist.

[3] Gaut BS (2015). Evolution is an experiment: assessing parallelism in crop domestication and experimental evolution: (Nei lecture, SMBE 2014, Puerto Rico). Mol Biol Evol.

[4] Sedivy EJ, Wu F, Hanzawa Y (2017). Soybean domestication: the origin, genetic architecture and molecular bases. New Phytol.

[5] Zhu G, Wang S, Huang Z, Zhang S, Liao Q, Zhang C (2018). Rewiring of the fruit metabolome in tomato breeding. Cell.

[6] Lin Z, Li X, Shannon LM, Yeh CT, Wang ML, Bai G (2012). Parallel domestication of the Shattering1 genes in cereals. Nat Genet.

[7] Fuller DQ, Denham T, Arroyo-Kalin M, Lucas L, Stevens CJ, Qin L (2014). Convergent evolution and parallelism in plant domestication revealed by an expanding archaeological record. Proc Natl Acad Sci U S A.

